# Supplementary material for: Cardiovascular health of women 10 to 20 years after placenta-related pregnancy diseases considering the possible effect of pentaerythrityl tetranitrate treatment during pregnancy on long-term maternal cardiovascular health (PAVA study)
Source: PLoS One. 2024 Oct 15;19(10):e0309177. doi: 10.1371/journal.pone.0309177 (PMC11478798; doi:10.1371/journal.pone.0309177)
Supplement: S1 Table — *Data are n (%) or median (25th-75th percentile). Number of subjects (N) is given if deviating from indicated group size. Significant results by Mann-Whitney-U-Test (p < 0.05) are highlighted in bold. p**comparing uneventful pregnancies vs. pregnancies complicated by PE/FGR without PETN intake; p***comparing uneventful pregnancies vs. pregnancies complicated by PE/FGR with PETN intake; PE, preeclampsia; FGR, fetal growth restriction; PETN, pentaerythritol tetranitrate; USCOM, Ultrasonic Cardiac Output Monitors; HR, heart rate; SV, stroke volume; SVI, stroke volume index; CO, cardiac output; CI, cardiac index; SVR, systemic vascular resistance; SVRI, systemic vascular resistance index; VPK, peak velocity of ventricular ejection; VTI, velocity time integral; MD, minute distance; ET, ejection time; FTc, flow time corrected; SVV, stroke volume variation; SMII, Smith Madigan Inotropy Index; VICORDER; AoPP, aortic pulse pressure; AoBP sys, aortic blood pressure systolic; AoBP dia, aortic blood pressure diastolic; MAP, mean arterial pressure; Aix, augmentation index; SEVR, subendocardial viability ratio; TPR, total peripheral resistance; FMS, flow mediated slowing; PWV, pulse wave velocity. (PDF) [file pone.0309177.s002.pdf]

Supplemental Table S1: Results of cardiovascular function analysis*					
	**Women with uneventful pregnancies (N=51)**	**Women with former PE/FGR without PETN (N=40)**	**p****	**Women with former PE/FGR with PETN (N=13)**	**p*****
**USCOM**					
HR(bpm)	67 (62–72)	65 (60–73)	0.499	72.5 (66–78.8)	0.119
SV(ml)	74 (61–82)	71 (64–80)	0.571	57.5 (54.3–63.8)	**0.003**
SVI(ml/m2)	37 (33–44)	37 (31–41)	0.387	31 (23.8–39)	**0.020**
CO(l/min)	4.7 (4.2–5.6)	4.4 (3.7–5.7)	0.420	4.2 (3.6–4.5)	**0.023**
CI(l/min/m2)	2.6 (2.2–2.9)	2.3 (2–2.9)	0.189	2.2 (1.9–2.5)	**0.030**
SVR(ds/cm5)	1,787 (1,469–2,046)	1,669 (1,478–2,398)	0.423	1,997(1,858–2,443)	**0.037**
SVRI(ds/cm5*m2)	3,267 (2,711–6,020)	5,090 (2,814–6,586)	0.211	5,840 (3,165–7,440.8)	0.074
VPK (m/s)	1.1 (1–1.3)	1.1 (1.0–1.3)	0.990	1 (0.9–1)	**0.022**
VTI (cm)	25 (21–27)	24 (23–28)	0.826	20 (19–23.8)	**0.006**
MD (m/min)	16.3 (14–18.7)	15 (12–19.3)	0.478	14 (12.4–16.4)	**0.044**
ET(%)	39 (37–42)	39 (35–41)	0.428	41.5 (37.5–44)	0.257
FTc(ms)	376 (360–388)	368 (347–389)	0.330	370 (348.5–393.8)	0.827
SVV(%)	23 (17–28)	24 (19–33)	0.199	23 (18.3–34.8)	0.528
SMII(W/m2)	1.5 (1.3–1.8)	1.5 (1.3–1.6)	0.653	1.2 (1.1–1.5)	**0.017**
**VICORDER**					
PWV(m/s)	5 (4–8)N=49	6 (4–8.8)	0.533	6 (4–8)	0.993
Aix	24 (19–31)	26 (19.3–29)	0.994	23 (19.5–29.5)	0.770
AoPP(mmHg)	64 (54–71)	64.5 (60–74.5)	0.364	63 (56–66.5)	0.802
AoBP sys(mmHg)	131 (119–144)	137 (123.5–148)	0.190	134 (124.5–149)	0.363
AoBP dia(mmHg)	68 (63–73)	68 (62–77.8)	0.514	74 (67.5–81)	**0.031**
MAP(mmHg)	95 (89–104)	97.5 (91–108.8)	0.198	101 (93.5–111.5)	0.095
SV(ml)	109 (97–127)	115 (99–133.3)	0.482	109 (96–114)	0.433
CO(l/min)	7 (6–8)	7 (7–9)	0.119	7 (6.5–8)	0.932
CI(l/min/m2)	4 (3–4)	4 (4–5)	0.173	4 (3–4.5)	0.750
SEVR(%)	158 (138–174)	146 (133.8–164.8)	0.171	151 (130.5–160.5)	0.301
TPR(PRU)	0.8 (0.7–1)N=49	0.8(0.7–1)	0.764	0.9 (0.8–1)	0.421
FMS(%)	16 (9.3–22.8)N=48	14 (10–21)	0.602	11.5 (7.3–21)	0.205

*Data are n (%) or median (25th-75th percentile). Number of subjects (N) is given if deviating from indicated group size. Significant results by Mann-Whitney-U-Test ($p<0.05$) are highlighted in bold. p**comparing uneventful pregnancies vs. pregnancies complicated by PE/FGR without PETN intake; p***comparing uneventful pregnancies vs. pregnancies complicated by PE/FGR with PETN intake; PE, preeclampsia; FGR, fetal growth restriction; PETN, pentaerythritol tetranitrate; USCOM, Ultrasonic Cardiac Output Monitors; HR, heart rate; SV, stroke volume; SVI, stroke volume index; CO, cardiac output; CI, cardiac index; SVR, systemic vascular resistance; SVRI, systemic vascular resistance index; VPK, peak velocity of ventricular ejection; VTI, velocity time integral; MD, minute distance; ET, ejection time; FT_c, flow time corrected; SVV, stroke volume variation; SMII, Smith Madigan Inotropy Index; VICORDER; AoPP, aortic pulse pressure; AoBP sys, aortic blood pressure systolic; AoBP dia, aortic blood pressure diastolic; MAP, mean arterial pressure; Aix, augmentation index; SEVR, subendocardial viability ratio; TPR, total peripheral resistance; FMS, flow mediated slowing; PWV, pulse wave velocity
