# Supplementary material for: Cardiovascular health of women 10 to 20 years after placenta-related pregnancy diseases considering the possible effect of pentaerythrityl tetranitrate treatment during pregnancy on long-term maternal cardiovascular health (PAVA study)
Source: PLoS One. 2024 Oct 15;19(10):e0309177. doi: 10.1371/journal.pone.0309177 (PMC11478798; doi:10.1371/journal.pone.0309177)
Supplement: S2 Table — *Data are n (%) or median (25th-75th percentile). Number of subject (N) is given if deviating from indicated group size. Significant results by Mann-Whitney-U-Test (p < 0.05) are highlighted in bold. p**comparing pregnancies complicated by PE/FGR without PETN intake vs. pregnancies complicated by PE/FGR with PETN intake; PE, preeclampsia; FGR, fetal growth restriction; PETN, pentaerythritol tetranitrate; USCOM, Ultrasonic Cardiac Output Monitors; HR, heart rate; SV, stroke volume; SVI, stroke volume index; CO, cardiac output; CI, cardiac index; SVR, systemic vascular resistance; SVRI, systemic vascular resistance index; VPK, peak velocity of ventricular ejection; VTI, velocity time integral; MD, minute distance; ET, ejection time; FTc, flow time corrected; SVV, stroke volume variation; SMII, Smith Madigan Inotropy Index; AoPP, aortic pulse pressure; AoBP sys, aortic blood pressure systolic; AoBP dia, aortic blood pressure diastolic; MAP, mean arterial pressure; Aix, augmentation index; SEVR, subendocardial viability ratio; TPR, total peripheral resistance; FMS, flow mediated slowing; PWV, pulse wave velocity; M, M-mode; 4CH, 4-chamber view; 2CH, 2-chamber view; LVEDD, left ventricular end-diastolic diameter; LVESD, left ventricular end-systolic diameter; LVPWD, left ventricular rear wall diameter; LA area, left atrial area; LA volume, left atrial volume; RVEDD, right ventricular end-diastolic diameter; RVESD, right ventricular end-systolic diameter; RA area, right atrial area; lVSD, interventricular septum diameter; LVEF, left ventricular ejection fraction; TAPSE, tricuspid annular plane systolic excursion; sPAP, systolic pulmonary arterial pressure; RVOT, right ventricular outflow tract; PH, pulmonary trunk diameter; IVC, inferior vena cava diameter; PVR, pulmonary vascular resistance; TRV, tricuspid regurgitant velocity; TVI RVOT, time-velocity integral of right ventricular outflow tract; E/A, E/A ratio; DCT, deceleration time; E/ med e´, E-wave velocity/ medial e [file pone.0309177.s003.pdf]

Supplemental Table S2: Results of cardiovascular function analysis*			
	**Women with former PE/FGR without PETN (N=40)**	**Women with former PE/FGR with PETN (N=13)**	**p****
**USCOM**			
HR$^{(bpm)}$	65 (60–73)	72.5 (66–78.8)	0.107
SV$^{(ml)}$	71 (64–80)	57.5 (54.3–63.8)	**0.007**
SVI$^{(ml/m2)}$	37 (31–41)	31 (23.8–39)	**0.077**
CO$^{(l/min)}$	4.4 (3.7–5.7)	4.2 (3.6–4.5)	0.234
CI$^{(l/min/m2)}$	2.3 (2–2.9)	2.2 (1.9–2.5)	0.280
SVR$^{(ds/cm5)}$	1,669 (1,478–2,398)	1,997(1,858–2,443)	0.222
SVRI$^{(ds/cm5*m2)}$	5,090 (2,814–6,586)	5,840 (3,165–7,440.8)	0.430
VPK $^{(m/s)}$	1.1 (1.0–1.3)	1 (0.9–1)	**0.032**
VTI $^{(cm)}$	24 (23–28)	20 (19–23.8)	**0.022**
MD $^{(m/min)}$	15 (12–19.3)	14 (12.4–16.4)	0.323
ET$^{(\%)}$	39 (35–41)	41.5 (37.5–44)	0.119
FTc$^{(ms)}$	368 (347–389)	370 (348.5–393.8)	0.731
SVV$^{(\%)}$	24 (19–33)	23 (18.3–34.8)	0.920
SMII$^{(W/m2)}$	1.5 (1.3–1.6)	1.2 (1.1–1.5)	**0.033**
**VICORDER**			
PWV$^{(m/s)}$	6 (4–8.8)	6 (4–8)	0.727
Aix	26 (19.3–29)	23 (19.5–29.5)	0.649
AoPP$^{(mmHg)}$	64.5 (60–74.5)	63 (56–66.5)	0.368
AoBP sys$^{(mmHg)}$	137 (123.5–148)	134 (124.5–149)	0.983
AoBP dia$^{(mmHg)}$	68 (62–77.8)	74 (67.5–81)	0.207
MAP$^{(mmHg)}$	97.5 (91–108.8)	101 (93.5–111.5)	0.463
SV$^{(ml)}$	115 (99–133.3)	109 (96–114)	0.137
CO$^{(l/min)}$	7 (7–9)	7 (6.5–8)	0.248
CI$^{(l/min/m2)}$	4 (4–5)	4 (3–4.5)	0.518
SEVR$^{(\%)}$	146 (133.8–164.8)	151 (130.5–160.5)	0.918
TPR$^{(PRU)}$	0.8(0.7–1)	0.9 (0.8–1)	0.489
FMS$^{(\%)}$	14 (10–21)	11.5 (7.3–21)	0.323
**Transthoracic echocardiography**			
HR$^{(bpm)}$	66.5 (63–75.8)$^{N=32}$	70.5 (64.8–77)$^{N=12}$	0.541
CO$^{(l/min)}$	3.4 (2.7–4.5)$^{N=33}$	4.4 (3.4–4.5)$^{N=12}$	0.189
SV$^{(ml)}$	53 (36–66.5)$^{N=33}$	57 (50.8–64.8)$^{N=12}$	0.568
LVEDD M$^{(mm)}$	45 (40–47.3)$^{N=34}$	44 (40.5–48)$^{N=12}$	0.688
LVEDD 4CH$^{(mm)}$	45 (42.8–47.3)$^{N=34}$	43 (37–45.5)$^{N=12}$	0.122
LVESD M$^{(mm)}$	26 (23–28.3)$^{N=34}$	28.5 (27–29.8)$^{N=12}$	**0.029**
LVESD 4CH$^{(mm)}$	28 (27–31)$^{N=34}$	27.5 (27–32)$^{N=12}$	0.743
LVPWD M$^{(mm)}$	9 (8–10)$^{N=34}$	8 (8–9)$^{N=12}$	0.177
LA area$^{(cm2)}$	17.5 (14–19)$^{N=34}$	14.5 (13–17.5)$^{N=12}$	**0.037**
LA volume$^{(ml)}$	45.5 (31.5–49.3)$^{N=34}$	36.5 (29.3–46.3)$^{N=12}$	0.225
RVEDD$^{(mm)}$	35 (31.8–37)$^{N=34}$	32 (29.3–33.8)$^{N=12}$	**0.025**
RVESD$^{(mm)}$	24 (20–26)$^{N=34}$	20.5 (19.3–22.5)$^{N=12}$	**0.043**
RA area$^{(cm2)}$	13 (11–15)$^{N=34}$	11 (10–12.8)$^{N=12}$	**0.029**
IVSD$^{(mm)}$	10 (9–11)$^{N=34}$	10 (9–11)$^{N=12}$	0.673
IVSD 4CH$^{(mm)}$	10 (9–11)$^{N=34}$	9 (8.3–10.3)$^{N=12}$	0.507
LVEF 2CH$^{(\%)}$	64.5 (60–67)$^{N=32}$	62 (60–67)$^{N=11}$	0.555
LVEF 4CH$^{(\%)}$	65 (59–67.5)$^{N=33}$	63 (58.8–66)$^{N=12}$	0.551
TAPSE$^{(mm)}$	23 (21–26.3)$^{N=30}$	20 (20–24)$^{N=9}$	0.203
sPAP$^{(mmHg)}$	19 (16–23)$^{N=25}$	16 (12.5–20.8)$^{N=6}$	0.291
RVOT$^{(mm)}$	28 (26–30.5)$^{N=33}$	25 (24–29)$^{N=11}$	0.078
PH$^{(mm)}$	21 (18.5–23)$^{N=21}$	19 (18–20.5)$^{N=9}$	0.137
IVC$^{(mm)}$)	13 (11–16)$^{N=33}$	13 (10.3–14)$^{N=12}$	0.568
PVR$^{(WU)}$	1.2 (0.6–1.5)$^{N=27}$	1.4 (1–1.7)$^{N=5}$	0.511
TRV$^{(m/s)}$	2.2 (2–2.4)$^{N=25}$	2 (1.8–2.3)$^{N=6}$	0.208
TVI RVOT$^{(cm)}$	17 (14–21.3)$^{N=30}$	17 (14.5–18)$^{N=8}$	0.661
E/A	1.5 (1.3–1.7)$^{N=33}$	1.5 (1–1.7)$^{N=12}$	0.603
DCT$^{(ms)}$	239 (194.5–265.5)$^{N=33}$	227 (185.3–256.3)$^{N=12}$	0.639
E/ med E	9.3 (7.2–11.5)$^{N=30}$	8.6 (7.8–10.3)$^{N=11}$	0.739
E/ lat E	6.6 (5.3–8)$^{N=27}$	6.4 (4.9–7.4)$^{N=7}$	0.901
Dia. Dysfunction	7 (24.1%)$^{N=29}$	2 (18.2%)$^{N=11}$	0.788

*Data are n (%) or median (25th-75th percentile). Number of subject (N) is given if deviating from indicated group size. Significant results by Mann-Whitney-U-Test ($p<0.05$) are highlighted in bold. p**comparing pregnancies complicated by PE/FGR without PETN intake vs. pregnancies complicated by PE/FGR with PETN intake; PE, preeclampsia; FGR, fetal growth restriction; PETN, pentaerythritol tetranitrate; USCOM, Ultrasonic Cardiac Output Monitors; HR, heart rate; SV, stroke volume; SVI, stroke volume index; CO, cardiac output; CI, cardiac index; SVR, systemic vascular resistance; SVRI, systemic vascular resistance index; VPK, peak velocity of ventricular ejection; VTI, velocity time integral; MD, minute distance; ET, ejection time; FT_c, flow time corrected; SVV, stroke volume variation; SMII, Smith Madigan Inotropy Index; AoPP, aortic pulse pressure; AoBP sys, aortic blood pressure systolic; AoBP dia, aortic blood pressure diastolic; MAP, mean arterial pressure; Aix, augmentation index; SEVR, subendocardial viability ratio; TPR, total peripheral resistance; FMS, flow mediated slowing; PWV, pulse wave velocity; M, M-mode; 4CH, 4-chamber view; 2CH, 2-chamber view; LVEDD, left ventricular end-diastolic diameter; LVESD, left ventricular end-systolic diameter; LVPWD, left ventricular rear wall diameter; LA area, left atrial area; LA volume, left atrial volume; RVEDD, right ventricular end-diastolic diameter; RVESD, right ventricular end-systolic diameter; RA area, right atrial area; IVSD, interventricular septum diameter; LVEF, left ventricular ejection fraction; TAPSE, tricuspid annular plane systolic excursion; sPAP, systolic pulmonary arterial pressure; RVOT, right ventricular outflow tract; PH, pulmonary trunk diameter; IVC, inferior vena cava diameter; PVR, pulmonary vascular resistance; TRV, tricuspid regurgitant velocity; TVI RVOT, time-velocity integral of right ventricular outflow tract; E/A, E/A ratio; DCT, deceleration time; E/ med e´, E-wave velocity/ medial e´-velocity- ratio; E/ lat e´, E-wave velocity/ lateral e´-velocity-ratio; Dia. Disfunction, existence of diastolic dysfunction
