# Supplementary material for: Cardiovascular health of women 10 to 20 years after placenta-related pregnancy diseases considering the possible effect of pentaerythrityl tetranitrate treatment during pregnancy on long-term maternal cardiovascular health (PAVA study)
Source: PLoS One. 2024 Oct 15;19(10):e0309177. doi: 10.1371/journal.pone.0309177 (PMC11478798; doi:10.1371/journal.pone.0309177)
Supplement: S1 File — (PDF) [file pone.0309177.s004.pdf]

Studienprotokoll

Präeklampsie assoziiertes vaskuläres Altern- Langzeitnachsorge und neue Präventionsstrategie

PAVA – Pilotstudie

Studienakronym:	PAVA-Pilotstudie
Protokollversion:	Erstversion vom 29.07.2019
Vertraulichkeitshinweis:	Der Inhalt vom vorliegenden Studienprotokoll ist vertraulich zu behandeln und darf ohne Zustimmung vom Studienleiter weder mündlich noch schriftlich an Unbeteiligte weitergegeben werden.

Inhaltsverzeichnis

1 Allgemeine Informationen ... 4
1.1 Beteiligte Personen, Institute und Gremien ... 4
1.2 Unterschriften ... 5
1.3 Zusammenfassung ... 5
1.4 Synopse ... 6
1.5 Ablaufdiagramm ... 7
1.6 Visitenplan ... 7
1.7 Verzeichnis Abkürzungen ... 9
2 Hintergrund ... 10
2.1 Ausgangssituation ... 10
2.2 Präeklampsie und das erhöhte Risiko für Herz-Kreislauf-Erkrankungen ... 11
2.3 Ansätze zur Behandlung der Endotheldysfunktionen bei Präeklampsie ... 12
3 Studienziele ... 13
4 Studiendesign und -beschreibung ... 14
4.1 Art der Studie ... 14
4.2 Art der Therapiezuordnung ... 14
4.3 Zahl und Art der Vergleichsgruppen ... 14
4.4 Umfang der Studie ... 14
4.5 Patientenrekrutierung ... 14
4.6 Zeitplan ... 15
5 Teilnehmende Einrichtungen ... 16
6 Auswahl der Patienten ... 17
6.1 Einschlusskriterien ... 17
6.2 Ausschlusskriterien ... 17
7 Studienablauf ... 17
7.1 Beschreibung der einzelnen Phasen des Studienablaufs ... 17
7.2 Aufklärung und Einwilligung ... 18
7.3 Randomisation ... 18

7.4 Nachbeobachtungsuntersuchungen ... 19
7.5 Beschreibung der einzelnen Visiten ... 19
7.6 Beschreibung der Labor- und anderen Untersuchungen und Methoden ... 19
7.7 Ende der Studienteilnahme ... 19
7.8 Vorzeitiges Ausscheiden eines Patienten aus der Studie (Abbruchkriterien) ... 19
8 Unerwünschte Ereignisse ... 20
8.1 Mögliche Komplikationen und/ oder Risiken ... 20
8.2 Erfassung und Dokumentation unerwünschter Ereignisse ... 20
9 Biometrie ... 20
10 Datenmanagement ... 20
11 Datenschutz ... 21
12 Ethische Belange, gesetzliche und administrative Regelungen ... 21
12.1 Deklaration von Helsinki und Gute klinische Praxis ... 21
12.2 Ethik-Kommission ... 21
12.3 Nachträgliche Änderungen ... 22
12.4 Patientenversicherung/ Probandenversicherung ... 22
12.5 Finanzierung ... 22
12.6 Abschluss und Publikation ... 22
13 Literatur ... 23
14 Anlagen ... 24

1 Allgemeine Informationen

1.1 Beteiligte Personen, Institute und Gremien

**Studienleiterin**		**Biometriker**	
**Name:**	Dr. med. Anna Multhaup	**Name:**	Dr. rer. pol. Thomas Lehmann
**Adresse:**	Klinik für Geburtsmedizin<br>Am Klinikum 1, 07747 Jena	**Adresse:**	Medizinische Statistik und Epidemiologie<br>Bachstraße 18, 07749 Jena
**Tel.:**	03641 9-329266	**Tel.:**	03641 9-396964
**E-Mail:**	friederike.weschenfelder@med.uni-jena.de	**E-Mail:**	thomas.lehmann@med.uni-jena.de
**Stellvertretende Studienleiterin**		**Datenmanagement und Projektmanagement**	
**Name:**	PD Dr. med. Tanja Groten	**Name:**	Yvonne Heimann, M.Sc.
**Adresse:**	Klinik für Geburtsmedizin<br>Am Klinikum 1, 07747 Jena	**Adresse:**	Klinik für Geburtsmedizin<br>Am Klinikum 1, 07747 Jena
**Tel.:**	03641 9-239206	**Tel.:**	03641 9-390868
**E-Mail:**	tanja.groten@med.uni-jena.de	**E-Mail:**	yvonne.heimann@med.uni-jena.de
**Labor**		**Labor**	
**Name:**	Apl. Prof. Dr.med. habil. R. Heller	**Name:**	PD Dr. Dr. Michael Kiehntopf
**Adresse:**	Institut für Molekulare Zellbiologie<br>Hans-Knöll-Str. 2, 07745 Jena	**Adresse:**	Institut für klinische Chemie und Laboratoriumsdiagnostik<br>Am Klinikum 1, 07747 Jena
**Tel.:**	03641 9-395633	**Tel.:**	03641 9-325001
**E-Mail:**	regine.heller@med.uni-jena.de	**E-Mail:**	michael.kiehntopf@med.uni-jena.de
**Kliniksdirektor**			
**Name:**	Prof. Dr.med. E. Schleußner		
**Adresse:**	Klinik für Geburtsmedizin<br>Am Klinikum 1, 07747 Jena		
**Tel.:**	03641 9-399200		
**E-Mail:**	ekkehard.schleussner@med.uni-jena.de		

1.3 Zusammenfassung

Die klinische Beobachtung, dass Frauen nach Präeklampsie ein hohes Risiko für früh einsetzende kardiovaskuläre Erkrankungen mit erhöhter krankheitsassoziierter Mortalität haben, hat zu der Hypothese geführt, dass der Endothelstatus dieser Frauen durch einen frühen Beginn des Alterns gekennzeichnet ist. Wir wollen den Zusammenhang zwischen Endothelalterung und Schwangerschaftsstörungen wie Präeklampsie, die von Endotheldysfunktionen dominiert werden, untersuchen. Gehen endotheliale Veränderungen der Schwangerschaft voraus und verursachen Präeklampsie und später ein beschleunigtes kardiovaskuläres Altern, oder löst Präeklampsie bei den Betroffenen erstmals eine vorzeitige endotheliale Alterung aus? Ziel dieser Studie ist es, die kardiovaskuläre Gesundheit von Frauen 10 bis 20 Jahre nach Präeklampsie zu untersuchen und mit der von Frauen mit ereignislosen Schwangerschaften zu vergleichen. Darüber hinaus wird die potenzielle langfristige endotheliale Schutzwirkung des NO-Donors Pentaerytrithyltetranitrat (PETN) untersucht.

Diese *in vivo* Studie wird *in vitro* durch die Analyse der Seneszenzinduktion an Endothelzellen nach Präeklampsie assoziiertem Stress erweitert. Zusätzlich wird die potentielle protektive Wirkung von PETN auf die stress-induzierte Seneszenz ausgewertet.

Das endgültige Ziel ist die Etablierung einer prospektiven Langzeitstudie über die Wirkung von PETN auf die Gefäßgesundheit von Frauen mit Präeklampsie, um zu beurteilen, ob die Behandlung der Endotheldysfunktion während der Schwangerschaft die Endothelalterung und damit die vorzeitige kardiovaskuläre Morbidität und Mortalität in Millionen von Frauen reduzieren könnte.

1.4 Synopse

**Titel der Studie**	Präeklampsie assoziiertes vaskuläres Altern – Langzeitnachsorge und neue Präventionsstrategie
**Kurzbezeichnung der Studie (Akronym)**	PAVA-Studie
**Studienleiterin**	Dr. Anna Multhaup
**Stellvertreter der Studienleiterin**	PD Dr. Tanja Groten
**Indikation**	Frauen nach Präeklampsie haben ein erhöhtes Risiko für vorzeitig einsetzende kardiovaskuläre Morbidität und Mortalität
**Studiendesign/ Methodik**	Eine monozentrische Analyse der vaskulären Gesundheit von Frauen 10–20 Jahre nach Präeklampsie.
**Ziele der klinischen Prüfung/ Zielstellung**	Bewertung der kardiovaskulären Gesundheit, insbesondere der Endothelgesundheit, von Frauen nach Präeklampsie im Vergleich zu Frauen ohne Präeklampsie, sowie der Vergleich von Frauen, die während der Schwangerschaft PETN eingenommen hatten, mit Frauen ohne Behandlungsversuch.
**Zielgrößen/ -kriterien/ Endpunkte**	Häufigkeiten von gefäßassoziierten Veränderungen und Erkrankungen (Hypertonie, Apoplex, Infarkt)
**Patientenzahl**	Rekrutierungsziel: 120 Frauen, davon 30 Kontrollen
**Einschlusskriterien**	• Teilnehmer der PETN-Pilotstudie von 2002–2008<br>• Kontrollen: ereignislosen Schwangerschaften von 2002–2008<br>• Patientinnen mit Präeklampsie mit PETN Einnahme vor 10–20 Jahren<br>• Kontrollen: Patientinnen mit Präeklampsie ohne PETN Einnahme vor 10–20 Jahren<br>• Schriftlicher Informed Consent<br>• Einlingsschwangerschaft
**Ausschlusskriterien**	• Patienten, die eine Teilnahme an der Studie ablehnen<br>• Unmöglichkeit der ambulanten Vorstellung
**Behandlungen/Verfahren, Behandlungsplan (inkl. Nachsorge)**	Die Untersuchung gliedert sich in zwei Abschnitte:<br>1. Basisdatenerhebung (Onlinebefragung oder schriftliche Form)<br>2. Ambulante Klinische Untersuchung

**Zeitplan (Studiendauer)**	patientenbezogen:<br>studienbezogen:<br>Gesamtdauer:	Einmalig 4 h für ambulante klinische Untersuchung<br>Rekrutierungsdauer: August 2019– August 2021<br>voraussichtlicher Abschluss einschl. Auswertung: Juni 2022
**Prüfzentren**	1	
**Statistische Methoden**		
**Finanzierung**	IZKF Clinician Scientist OrganAge Programm, MGFG Forschungsgeld	

1.5 Ablaufdiagramm

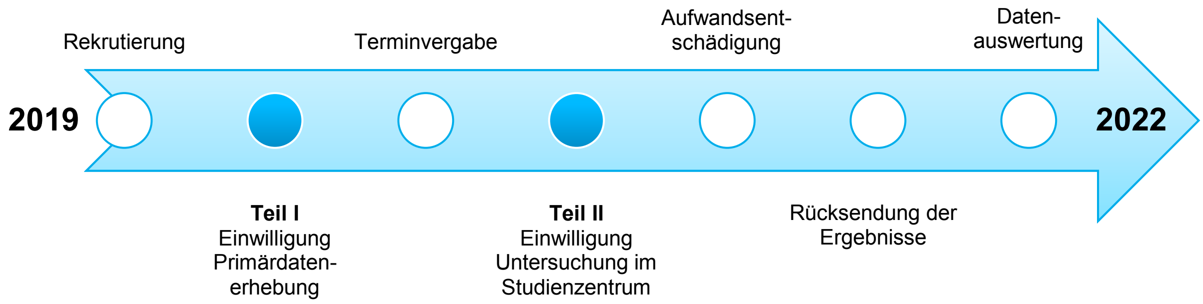

Abbildung 1: Arbeitsplan

1.6 Visitenplan

Tabelle 1: Parameter der kardiovaskulären Gesundheitsanalyse

**Teil I: Primärdatenerhebung**		
Studieninformation	Schriftliche Einwilligung	
Fragebogen	Demographische Daten	Alter, Ausbildung, Beruf, Einkommen, Familienstatus, Anzahl der Schwangerschaften, Anzahl der Kinder, Erkrankungen, Operationen, Medikamentenanamne-se, Raucher ja/nein, Drogen-/Alkohlkonsum, körperli-che Aktivität
**Teil II: Untersuchung**		
Aufklärung der klinischen Untersuchungen	Schriftliche Einwilligung zu klinischen Untersuchung	
Körperliche Untersuchung	Größe (in cm)	Erhebung mittels Stadiometer mit variabler Messlatte
	Gewicht (in kg)	Erhebung mit leichter Bekleidung auf einer digitalen geeichten medizinischen Personenwaage

	Body-Mass-Index (kg/m2)	Berechnung anhand Gewicht und Körpergröße
	Blutdruck (mmHg)	Gemessen von der Brachialarterie mit Manschette durch Quecksilber-Blutdruckmessung
Kardiale Messungen	Herzzeitvolumen (CO)	Uscom
Vaskuläre Messungen	Karotis intima media Dicke (CIMT)	Using Toshiba ultrasound system in high resolution B-mode
	Totaler peripherer Wider-stand (TVR)	Uscom
	Blutdruck (BP)	VICORDER®
	Flussvermittelte Gefäßdi-latation (FMD)	VICORDER®
	Kritische Flicker-Frequenz (CFF)	HEPAtonormTM
	Pulswellengeschwindig-keit (PWV)	Mobil-O-Graph 24h PWA Monitor und Hypertension Management Software Client-Server®, VICORDER®
	Augmentation index	
Serumanalyse	Basis Labor[1]	
	Multiplex Analyse	„Human vascular inflammation panel“
		„Human angiogenesis panel“
		„Adhesion molecule panel“

[1]*Auflistung der zu erhebenden Laborparameter:*

ASAT, ALAT, Natrium, Kalium, Kreatinin, GFR, Gerinnung (Quick, INR, aPTT), Blutbild (Leukozyten, Erythrozyten, Hämoglobin, Hämatokrit, MCV, MCH, MCHC, Thrombozyten, RDW), HDL, LDL, Trigly-ceride, TSH-Stufendiagram, C-Reactive Protein (CRP)

„Human vascular inflammation panel”:

Myoglobin, Calprotectin (MRP8/14), Lipocalin A (NGAL), CRP, MMP-2, Osteopontin (OPN), Myelo-peroxidase (MPO), Serum Amyloid A, IGFBP-4, ICAM-1 (CD54), VCAM-1 (CD106), MMP-9, Cystatin C

„Human angiogenesis panel”:

IL-6, Angiopoietin-1, Angiopoietin-2, EGF, FGF-basic, CXCL8 (IL-8), PECAM-1 (CD31), PlGF, VEGF, TNF-α

„Adhesion molecule panel“:

ICAM-1 (CD54), ICAM-2 (CD102), ICAM-3 (CD50), VCAM-1 (CD106), PECAM-1 (CD31), ALCAM-1 (CD166), EpCAM (CD326), NCAM (CD56), E-selectin, P-selectin, L-selectin, PSGL-1, and CD44

Tabelle 2: Ablauf Teil II Untersuchungen

Uhrzeit	Was?	Wer?
8.00	• Aufnahme im SAP<br>• Begrüßung, Aufklärung/ Einwilligung	Aufnahme<br>Arzt
8.30	• Anamnese<br>• Blutabnahmen<br>• körperliche Untersuchung	Hebamme/ Schwester
9.30	HEPAtonormTM (CFF)	Arzt
9.45	USCOM (CO, SVR)	Arzt
10.00	Carotis initma media Dicke	Arzt
10:30	Mobilograph (PWV, Augmentationsindex)	Arzt
10:45	VICORDER® (FMD, PWV, Augmentationsindex)	Arzt
11.00	Ende	

1.7 Verzeichnis Abkürzungen

Tabelle 3: Verzeichnis Abkürzungen

**PETN**	Pentaerytrithyltetranitrat
**PE**	Präeklampsie
**IUGR**	Intrauterine Wachstumsretardierung
**BMI**	_body mass index_, Körpermasseindex
**PWV**	_pulse wave velocity_, Pulswellengeschwindigkeit
**AIx**	Augmentationsindex
**FMD**	_flow mediated dialation_, Flussvermittelte Gefäßdilatation
**CIMT**	_carotis initma media thickness_, Carotis-Intima-Media-Dicke
**CFF**	_critical flicker frequency_, kritische Flickerfrequenz
**TVR**	_total vascular resistance_, totale periphere Widerstand
**CO**	_cardiac output,_ Herzzeitvolumen
**CVD**	_cardiovaskular disease_, Herz-Kreislauf-Erkrankungen
**PETN**	Pentaerytrithyltetranitrat
**PE**	Präeklampsie
**IUGR**	Intrauterine Wachstumsretardierung
**BMI**	_body mass index_, Körpermasseindex

**PWV**	*pulse wave velocity*, Pulswellengeschwindigkeit
**AIx**	Augmentationsindex
**FMD**	*flow mediated dialation*, Flussvermittelte Gefäßdilatation
**CIMT**	*carotis initma media thickness*, Carotis-Intima-Media-Dicke
**CFF**	*critical flicker frequency*, kritische Flickerfrequenz

2 Hintergrund

2.1 Ausgangssituation

Nach Angaben der Weltgesundheitsorganisation sind Herz-Kreislauf-Erkrankungen (CVD) für 30 % der Todesfälle weltweit verantwortlich (Sepulveda, Palomo, & Fuentes, 2017). Sie sind die häufigste Todesursache und die häufigste Morbidität in Deutschland; mit entsprechend hoher finanzieller Belastung (Bundesamt, 2018). Organalterung ist der größte Risikofaktor für Erkrankungen des Menschen. Der Alterungsprozess ist gekennzeichnet durch eine Funktionseinschränkung von Zellen und Gewebe und die verminderte Fähigkeit, angemessen auf Umwelteinflüsse wie metabolischen Stress und verminderte Sauerstoffversorgung zu reagieren. Die Alterung der Gefäße ist Hauptrisikofaktor für die Entwicklung von CVD (Regina et al., 2016).

Während der Schwangerschaft werden umfangreiche Anpassungen im Herz-Kreislauf-System den erhöhten Stoffwechselanforderungen von Mutter und Fetus gerecht. Das Plasmavolumen und das Herzzeitvolumen erreichen nach 32 Schwangerschaftswochen ein Maximum von 40–50 % über dem Ausgangswert, indem das Schlagvolumen auf 1,8 l/min und die Herzfrequenz um 15–20 Schläge/min erhöht werden. Weiterhin nehmen atrialer und ventrikulärer Durchmesser zu, während der systemische und pulmonale Gefäßwiderstand fällt (Regitz-Zagrosek et al., 2018). Schwangerschaft ist somit ein definierter kardiovaskulärer Belastungstest, und eine Dekompensation des kardiovaskulären Systems ist mit plazentaassoziierten Schwangerschaftskomplikationen wie Präeklampsie und fetaler Wachstumsretardierung verbunden (Orabona et al., 2017).

Präeklampsie (PE) ist definiert als die Entwicklung von Bluthochdruck und Proteinurie nach der 20. Schwangerschaftswoche bei einer zuvor normotensiven Frau. Es handelt sich um eine Multiorganerkrankung, die durch eine generalisierte endotheliale Dysfunktion der Mutter zu einem systemischen Entzündungszustand führt, der Hypertonie und Hyperkoagulopathie bedingt und in Fehlperfusion der Endorgane mit Gehirn-, Leber- und Nierenbeteiligung endet. Weltweit erkranken jedes Jahr mehr als vier Millionen Frauen (Steegers, von Dadelszen, Duvekot, & Pijnenborg, 2010).

Tatsache ist, dass Mütter mit vorbestehender endothelialer Dysfunktion (z. B. Hypertonie, Lupus erythematodes, Anti-Phospholipid-Antikörper-Syndrom) ein höheres Risiko haben, Präeklampsie zu entwickeln (Steegers et al., 2010). Darüber hinaus zeigen akkumulierende Studien, dass Frauen mit Schwangerschaftshypertonie oder Präeklampsie im späteren Erwachsenenalter ein erhöhtes Risiko

für Hypertonie, Schlaganfall und ischämische Herzerkrankungen aufweisen (Black et al., 2016). Aktuelle Daten zeigen zudem, dass diese Frauen ein über dreifach erhöhtes Risikos für vaskuläre Demenz haben (Basit, Wohlfahrt, & Boyd, 2018).

Dementsprechend besteht eine eindeutige Korrelation zwischen kardiovaskulären Erkrankungen, Gefäßalterung, -dysfunktion und Präeklampsie. Es bleibt jedoch unbeantwortet, ob es Frauen mit einem vorkonditionierten Gefäßzustand sind, die später Präeklampsie und CVD entwickeln, oder ob Präeklampsie zu einer langfristigen Schädigung und einer beschleunigten Alterung des Herz-Kreislauf-Systems führt, die schließlich CVD verursacht. Die Beantwortung dieser Frage ist für betroffene Frauen von entscheidender Bedeutung. Wenn Präeklampsie der erste Schlag ist, der zu einem Endothel mit akzellerierter Alterung führt, würde die Behandlung der Endotheldysfunktion während der Schwangerschaft zu einer Reduzierung der Präeklampsie und folglich zu einem geringeren Risiko für CVD im späteren Leben führen. Wenn Präeklampsie durch vorgeschädigtes Endothel bedingt ist, kann bereits vor Beginn der Schwangerschaft ein Risiko für betroffene Frauen erkannt werden, eine Behandlung begonnen und Präeklampsie und wahrscheinlich auch eine spätere CVD abgemildert werden. Um diese Frage zu klären, ist eine Langzeitbeobachtung des Gefäß- und Endothelzustands während des gesamten Schwangerschaftsverlaufs und danach durch randomisierte kontrollierte Studien erforderlich, in denen die Auswirkungen von Endothelzielbehandlungen auf die Entwicklung von Präeklampsie und fetaler Wachstumsretardierung während der Schwangerschaft verglichen werden.

Ziel dieser Pilotstudie ist es, eine Reihe von Untersuchungen durchzuführen, mit denen sich ein spezifisches Gefäßprofil identifizieren lässt, das mit Präeklampsie und dem Risiko einer späteren CVD verbunden ist. Darüber hinaus wird eine mögliche Änderung des Risikoprofils durch das endothelprotektive Medikament Pentaerytrithyltetranitrat (PETN) bei Frauen mit einem Risiko für Gefäßkomplikationen untersucht. Wir werden den kardiovaskulären Status von Frauen, die während der Schwangerschaft PETN einnahmen, mit Kontrollen vergleichen. Zusätzlich wird die Wirkung von PETN auf die Endothelalterung *in vitro* untersucht.

2.2 Präeklampsie und das erhöhte Risiko für Herz-Kreislauf-Erkrankungen

Während angenommen wird, dass eine unkomplizierte Schwangerschaft die vaskuläre Compliance vorübergehend verbessert (Orabona et al., 2017), wurde Präeklampsie als unabhängiger Risikofaktor für eine subklinische Arteriosklerose noch über 10 Jahre nach PE nachgewiesen (Garovic et al., 2017). Eine Metaanalyse von Studien ergab zudem, dass Frauen mit PE zum Zeitpunkt der Erkrankung, sowie 10 Jahre danach, eine signifikant höhere Carotis-Intima-Media-Dicke (CIMT) aufwiesen (Milic et al., 2017). CIMT gilt als Prädikator für kardiovaskuläre Ereignisse (Peters, den Ruijter, Grobbee, & Bots, 2013). Frauen mit Bluthochdruck in ihrer ersten Schwangerschaft haben ein erhöhtes Rezidivrisiko in nachfolgenden Schwangerschaften; die ESC-Richtlinie korreliert sogar: je früher der Bluthochdruck einsetzt, desto höher ist das Rezidivrisiko (Regitz-Zagrosek et al., 2018). Diese

Beobachtungen weisen auf eine irreparable endotheliale Dysfunktion hin. Bestätigend berichteten Melchoirre et al., dass 40 % der Frauen mit *early-onset* Präeklampsie innerhalb von 1 bis 2 Jahren nach der Schwangerschaft eine essentielle Hypertonie entwickelten (Melchiorre, Sutherland, Liberati, & Thilaganathan, 2011).

Arterielle Steifheit, welche als unabhängiger Prädikator für kardiovaskuläre Morbidität und frühe kardiovaskuläre Mortalität gilt, ist ebenfalls schon vor der klinischen Phase der schwangerschafts-induzierten Hypertonie erhöht (Orabona et al., 2017). Sie kann durch die Pulswellengeschwindigkeit (PWV) quantifiziert werden, die sich als spezifisches und hochempfindliches Instrument zur Detektion von Präeklampsie in der Schwangerschaft erwiesen hat. Als sensitiverer Frühindikator für die arterielle Steifheit wird ebenso der Augmentationsindex (AIx) verstanden (Foo, McEniery, Lees, Khalil, & International Working Group on Maternal, 2017).

Tiralongo et al. beobachteten, dass ein anhaltend hoher peripherer Gefäßwiderstand und eine fehlende Erhöhung des Herzzeitvolumens vor der 16. Schwangerschaftswoche ebenfalls ein Hinweis auf die Entwicklung einer Präeklampsie waren (Tiralongo et al., 2015). MeLaughlin et al. stellten in Übereinstimmung mit Anderen fest, dass der totale Gefäßwiderstand der Mutter (TVR) der beste einzelne prädiktive Faktor für die Entwicklung von Hypertonie in der Schwangerschaft war (McLaughlin, Zhang, Lye, Parker, & Kingdom, 2018). Vasapollo beschrieb, dass der TVR der beste unabhängige Prädikator für mütterliche und fetale Komplikationen während der Schwangerschaft sei. Eine Reduktion der TVR um > 20 % bei hypertensiven Patientinnen schien schwerwiegende Komplikationen zu reduzieren (Vasapollo et al., 2012). Nach der Geburt, hatten Frauen mit PE im Vergleich zu Frauen mit unauffälligen Schwangerschaften immer noch erhöhte TVR- und Blutdruckwerte (Valensise et al., 2016). Das Fortbestehen einer endothelialen Dysfunktion kann die Verbindung zwischen Plazentationsstörung und dem kardiovaskulären Risiko im späteren Leben darstellen. Patientinnen mit früher PE zeigten in Studien über eine signifikante Minderung der Flussvermittelten Dilatation (FMD) Hinweise auf anhaltende Mikrozirkulationsstörung (Orabona et al., 2017).

Obwohl verschiebe Einzeluntersuchungen stattfanden gibt es bisher keine umfassende Bewertung des langfristigen kardiovaskulären Status von Frauen nach Präeklampsie.

2.3 Ansätze zur Behandlung der Endotheldysfunktionen bei Präeklampsie

Die Bedeutung von oxidativem Stress und endothelialer Dysfunktion in der Pathophysiologie der Präeklampsie hat zur Entwicklung von therapeutischen Ansätzen mit Ziel der Wiederherstellung des Redoxgleichgewichts geführt, wie Vitamine, Protonenpumpenhemmer und Aspirin. Bisher konnte nur für Aspirin ein signifikanter Einfluss nachgewiesen werden. Eine kürzlich durchgeführte internationale multizentrische RCT zeigte eine signifikante Verringerung der *early-onset* PE durch die tägliche Gabe von Aspirin, wenn sie vor der 16. Schwangerschaftswoche bei Personen mit erhöhtem Risikoprofil

begonnen wurde. Es konnte jedoch kein signifikanter Effekt auf die spät einsetzende (*late-onset*) Präeklampsie erzielt werden (Rolnik et al., 2017).

Wir haben die Wirksamkeit des NO-Donors Pentaeritrithyltetranitrat (PETN) zur Sekundärprävention der fetalen Wachstumsretardierung, der PE und der Frühgeburt bei Schwangerschaften mit einem Risiko für plazentabedingte Schwangerschaftskomplikationen in einer prospektiven, randomisierten, Placebo kontrollierten, doppelblinden Studie von 2002–2008 in der Klinik für Geburtsmedizin in Jena untersucht. In dieser Pilotstudie konnte PETN das Risiko einer ausgeprägten fetalen Wachstumsretardierung und perinatalen Tod um 39 % senken und erzielte des Weiteren eine 70 %-ige Verringerung der Frühgeburt vor der vollendeten 32. SSW (bereinigter OR 0,204; 95 % CI 0,052–0,801) (Bowkalow et al., 2018; Schleussner et al., 2014). Die Gesamtzahl der Patientinnen, die eine PE entwickelten, unterschied sich nicht zwischen den Gruppen, der Beginn war jedoch verzögert und der Schweregrad verringert. Auf der Grundlage dieser Pilotstudie führen wir derzeit eine multizentrische, randomisierte, Placebo kontrollierte Studie durch, um den nachgewiesenen klinischen Effekt auf die Wachstumsretardierung zu bestätigen. Es ist eine Anzahl von 324 Patientinnen vorgesehen, die in 14 Zentren in Deutschland rekrutiert werden (GR1955, 4-1; NCT: NCT03669185).

Neben seiner gefäßerweiternden Wirkung scheint PETN, wahrscheinlich aufgrund seines Einflusses auf HO-1, das Fortschreiten der Arteriosklerose zu verhindern und die Endothelfunktion durch antiproliferative und antiapoptotische Mechanismen zu verbessern. Vor kurzem wies unsere Gruppe eine signifikante Reduktion der stressinduzierten endothelialen Dysfunktion durch PETN *in-vitro* nach. Durch Serumentzug bedingte Veränderungen der Proliferation, Migration und Angiogenese von Endothelzellen wurden nach PETN Gabe signifikant reduziert. Somit konnte die Schutzwirkung von PETN auf Endothelzellen *in vitro* verifiziert und ein Konzept aufgestellt werden, wie die PETN-Behandlung die endotheliale Resistenz gegen schwangerschaftsbedingten Stress *in-vivo* stabilisieren könnte (Teichert V., 2018).

3 Studienziele

Ziel dieser Studie ist es, die Gefäßgesundheit von Frauen 10 bis 20 Jahre nach Präeklampsie im Vergleich zu Frauen mit komplikationslosen Schwangerschaften zu analysieren und darüber hinaus die potenzielle Schutzwirkung von PETN auf die Endothelalterung zu bewerten.

Drei Arbeitspakete sind vorgesehen:

1. Vergleich der vaskulären Gesundheit von Patientinnen nach Präeklampsie mit der von Frauen mit komplikationsfreien Schwangerschaften 10–20 Jahre nach der Schwangerschaft.
2. Analyse eines möglichen Langzeiteffekts der PETN-Behandlung während der Schwangerschaft anhand eines Gefäßgesundheitsvergleichs von Patientinnen, die von 2002 bis 2008 im Rahmen des PETN-Pilotstudie PETN erhalten hatten, oder dieses im Rahmen eines personalisierten Therapieversuchs bekamen, mit denjenigen die Placebo oder keine Therapie erhielten.
3. *In-vitro*-Analyse der Endothelzellseneszens nach Präeklampsie induziertem Stresseffekt, sowie des möglichen Schutzeffekts von PETN auf die endotheliale Zellalterung.

4 Studiendesign und -beschreibung

4.1 Art der Studie

Es handelt sich um eine klinische Nachuntersuchung der kardiovaskulären, vor allem endothelialen Gesundheit, von den Frauen die von 2002–2008 an der PETN-Pilotstudie teilnahmen, sowie die vergleichende Untersuchungen von Frauen ohne Medikation oder ohne Schwangerschaftskomplikationen in der gleichen Zeit.

4.2 Art der Therapiezuordnung

N.A.

4.3 Zahl und Art der Vergleichsgruppen

Siehe 4.5 Tabelle 4.

4.4 Umfang der Studie

Die Studie wird unizentrisch durchgeführt und soll insgesamt 120 Frauen einschließen.

4.5 Patientenrekrutierung

Kohortenrekrutierung Arbeitspaket 1:

Patientinnen mit Präeklampsie vor 10 bis 20 Jahren werden über die SAP-Datenbank herausgesucht. Die Patientinnen werden mit der nächsten, einwilligenden Patientin gematched, die ohne Schwangerschaftskomplikationen entbunden hatte. Es werden nur Einlingsschwangerschaften berücksichtigt, die zwischen der 24. und 42. Schwangerschaftswoche geboren wurden.

Kohortenrekrutierung Arbeitspaket 2:

Patientinnen mit und ohne PETN-Behandlung werden aus den 111 Patientinnen rekrutiert, die von 2002 bis 2008 an der PETN-Pilotstudie teilgenommen hatten. Zusätzlich werden Patientinnen rekrutiert, die eine PETN-Behandlung als personalisierten Therapieversuch bekamen. Diese werden durch Patientinnen ohne Behandlung gematched. Das Matching umfasst die Indikation zur Behandlung, die Schwangerschaftswoche der Diagnose und die Perzentile des fetalen Wachstums.

Tabelle 4: Übersicht der Kohorten

Kohorten	Zielgruppe	Kontrollen	Rekrutierungsziel (120)
**1**	Patientinnen mit Präeklampsie, welche zwischen 1999–2009 an der Uniklinik Jena betreut wurden	Matched Patientinnen mit komplikationsfreien Schwangerschaften	(30:30)
**2a**	Patientinnen der PETN Pilotstudie, mit PETN (54)	Patientinnen der PETN-Pilotstudie mit Placebo (57)	(20:20)
**2b**	Patientinnen, welche als individuellen Therapieversuch PETN erhielten (25)	Patientinnen gematched durch Schwangerschaftskompliaktionen, welche kein PETN erhielten	(10:10)

4.6 Zeitplan

Die Studiendauer ist dem IZKF Stipendium Clinician Scientist OrganAge angepasst und ist für drei Jahre (01.07.2019–30.06.2022) vorgesehen. Dabei sollen die Rekrutierungen 2019 erfolgen, die klinischen Untersuchungen 2019–2021 und die Datenauswertung 2020–2022 stattfinden (siehe Abbildung 2).

Die Patientinnen werden angeschrieben und willigen ggf. zur Studienteilnahme ein. Es wird weiteres Informationsmaterial zugeschickt und ein Termin zur klinischen Untersuchung vereinbart. Eine einmalige klinische Vorstellung und Untersuchung findet statt. Dieser Prozess sollte innerhalb von jeweils 4 Monaten erfolgen.

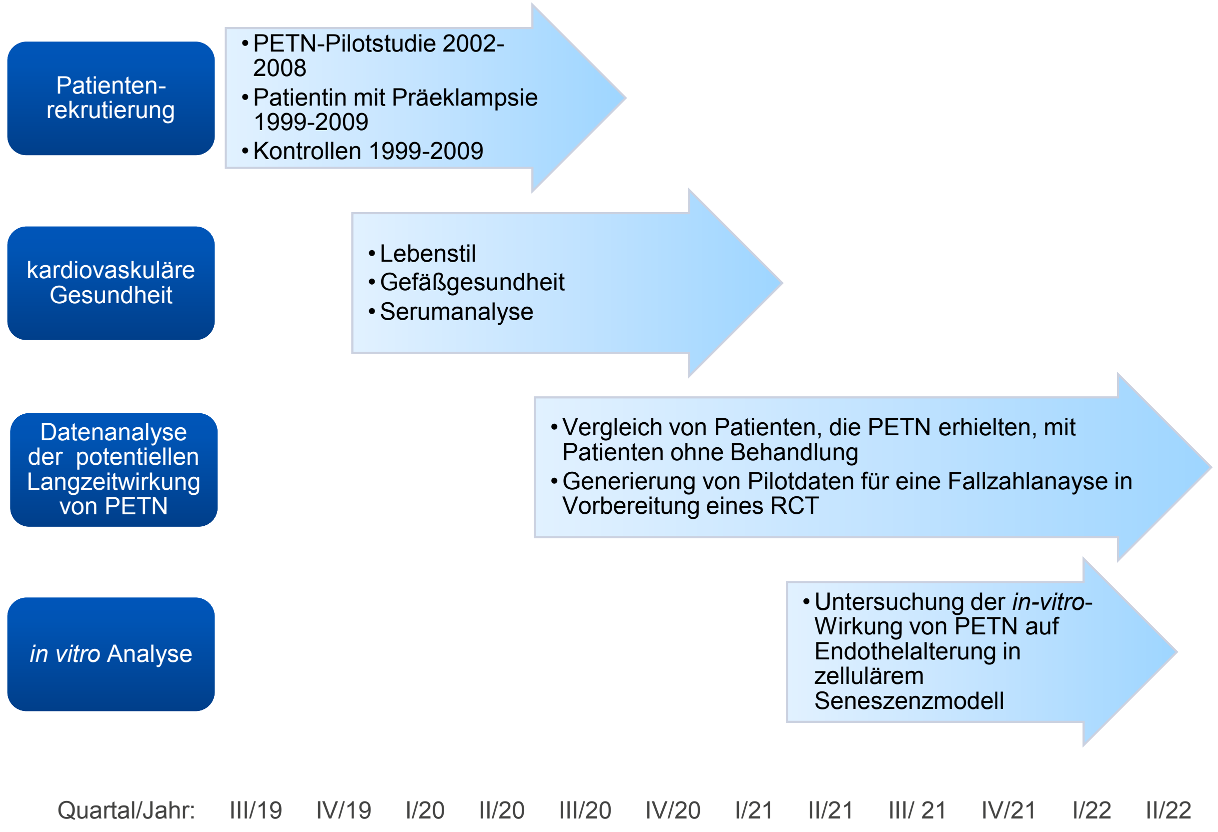

Abbildung 2: Zeitplan

5 Teilnehmende Einrichtungen

Klinik/Institution:	Klinik für Geburtsmedizin
	Friedrich-Schiller-Universität Jena
	Universitätsklinikum Jena
	Am Klinikum 1
	07747 Jena
Telefon-Nummer:	03641 9-329201
E-Mail:	Gabriele.Schack@med.uni-jena.de

6 Auswahl der Patienten

Siehe 4.5 Patientenrekrutierung.

6.1 Einschlusskriterien

- Teilnehmer der PETN-Pilotstudie von 2002–2008
- Kontrollen: ereignislosen Schwangerschaften von 2002–2008
- Patientinnen mit Präeklampsie mit PETN Einnahme vor 10–20 Jahren
- Kontrollen: Patientinnen mit Präeklampsie ohne PETN Einnahme vor 10–20 Jahren
- Schriftlicher Informed Consent
- Einlingsschwangerschaft

6.2 Ausschlusskriterien

- Patienten, die eine Teilnahme an der Studie ablehnen
- Unmöglichkeit der ambulanten Vorstellung

7 Studienablauf

7.1 Beschreibung der einzelnen Phasen des Studienablaufs

Nach Erhalt der Patientendaten (Einschlusskriterien, Adresse) aus dem SAP oder der Scan-Akte wird ein Anschreiben mit Anfrage der Kontaktaufnahme für Studienzwecke gestellt (siehe Anhang Anschreiben). Falls die Patientin an der Studie interessiert ist, wird ein weiterer Brief mit Informationsmaterial (siehe Anhang Infomaterial), Fragebogen (siehe Anhang Fragebogen) und Einwilligung zur Studienteilnahme (siehe Anhang Studieneinwilligung) verschickt. Telefonisch wird ein Termin zur klinischen Untersuchung vereinbart.

Bei Eintreffen in unserer Ambulanz erfolgt das Einsammeln, ggf. gemeinsames Ausfüllen des Fragebogens, sowie die Aufklärung und schriftliche Einwilligung zum klinischen Teil der Studie.

Die Aufklärung jeder Patientin über Wesen, Bedeutung, Ziele, mögliche Risiken, erwartete Vorteile, Tragweite und sonstige Aspekte der klinischen Untersuchung erfolgt durch ein Gespräch zwischen der Studienleiterin und der Patientin. Die Patientin erhält die schriftliche Patienteninformation. Nach der Aufklärung erhält jede Patientin ausreichend Zeit und Gelegenheit offene Fragen zu klären und über ihre Teilnahme zu entscheiden.

Jede Patientin unterzeichnet und datiert ihre Einwilligung in die Teilnahme an der Studie eigenhändig schriftlich auf der Einwilligungserklärung. Die Patientinnen werden explizit über Zweck und Umfang der Erhebung und die Verwendung der Daten, insbesondere von Gesundheitsdaten, informiert.

Ist eine Patientin nicht in der Lage eigenhändig die Einwilligung zu unterzeichnen, wird sie nicht in die Studie eingeschlossen.

Ein Exemplar der unterschriebenen Einwilligungserklärung (Kopie oder 2. Original) wird der Patientin ausgehändigt, das andere verbleibt im Prüfzentrum.

Die Patientin kann jederzeit und ohne Angabe von Gründen die Einwilligung zurückziehen und die Untersuchungen abbrechen.

Es folgen die in 7.1.3 aufgeführten Untersuchungen. Die Übergabe der Anfahrtsentschädigung erfolgt nach der letzten klinischen Untersuchung.

Das reguläre Ende der Studienteilnahme für jede Teilnehmerin ist nach Abschluss der Untersuchungen, wobei das Zusenden der persönlichen Untersuchungsergebnisse per Post nach Analyse später erfolgt.

Die Teilnahme an der klinischen Prüfung ist freiwillig. Jede Teilnehmerin hat das Recht, jederzeit auf eigenen Wunsch vorzeitig und ohne Angabe von Gründen aus der Studie auszuscheiden (Einwilligung wird zurückgezogen) oder sich für einen Untersuchungsabbruch (keine weitere Untersuchung) zu entscheiden. Die Patientin wird in solchen Fällen gebeten, den Abbruchgrund zu nennen, wird jedoch darauf hingewiesen, dass sie dies nicht tun muss. Der Zeitpunkt der Rücknahme der Einwilligung ist zu dokumentieren.

Bei vorzeitigem Abbruch der Untersuchungen wird die Patientin gefragt, ob die ggf. schon erhobenen Messungen oder abgenommen Blutproben analysiert werden dürfen. Dies wird dokumentiert und von der Patientin unterzeichnet.

Es ist keine erneute Nachbeobachtung vorgesehen.

7.2 Aufklärung und Einwilligung

Siehe angefügte Patienteninformation und Einwilligungserklärung.

7.3 Randomisation

N.A.

7.4 Nachbeobachtungsuntersuchungen

N.A.

7.5 Beschreibung der einzelnen Visiten

Siehe 1.6 Tabelle 2.

7.6 Beschreibung der Labor- und anderen Untersuchungen und Methoden

Die geplante Blutanalyse erfolgt über das Routinelabor des Institut für Klinische Chemie und Laboratoriumsdiagnostik. Die Multiplexanalysen aus dem Patientinnenserum werden durch die Antragstellerin in Kooperation mit dem Labor von Frau Prof. R. Heller durchgeführt. Alle weiteren Untersuchungen erfolgen in der Ambulanz für Geburtsmedizin. Die Untersuchungen sind in 1.6 Tabelle 1 zu entnehmen. Weitere Information zu den Geräten sind als Anhang zusammengestellt.

7.7 Ende der Studienteilnahme

Das reguläre Ende der Studienteilnahme für jede Teilnehmerin ist nach Abschluss der Untersuchungen, wobei das Zusenden der persönlichen Untersuchungsergebnisse per Post nach Analyse später erfolgt.

7.8 Vorzeitiges Ausscheiden eines Patienten aus der Studie (Abbruchkriterien)

Die Teilnahme an der klinischen Prüfung ist freiwillig. Jede Teilnehmerin hat das Recht, jederzeit auf eigenen Wunsch vorzeitig und ohne Angabe von Gründen aus der Studie auszuscheiden (Einwilligung wird zurückgezogen) oder sich für einen Untersuchungsabbruch (keine weitere Untersuchung) zu entscheiden. Die Patientin wird in solchen Fällen gebeten, den Abbruchgrund zu nennen, wird jedoch darauf hingewiesen, dass sie dies nicht tun muss. Der Zeitpunkt der Rücknahme der Einwilligung ist zu dokumentieren.

Bei vorzeitigem Abbruch der Untersuchungen wird die Patientin gefragt, ob die ggf. schon erhobenen Messungen oder abgenommen Blutproben analysiert werden dürfen. Dies wird dokumentiert und von der Patientin unterzeichnet.

8 Unerwünschte Ereignisse

8.1 Mögliche Komplikationen und/ oder Risiken

- Komplikationen oder Nebenwirkungen der Blutabnahme: Hämatom, Schmerz, Infektion
- Komplikation der Messung: Schmerz u./o. Petechien (VICORDER®)

8.2 Erfassung und Dokumentation unerwünschter Ereignisse

Eine Erfassung unerwünschter Ereignisse erfolgt im Rahmen der Dokumentation.

9 Biometrie

Die Ergebnisse dieser Pilotuntersuchung sollen die Grundlage für die Fallzahlplanung einer im Anschluss geplanten prospektiven Studie schaffen. Die hier gewählte Fallzahl ergibt sich aus den gewählten Kollektiven. Die Kontrollgruppen wurden in gleicher Gruppenstärke gewählt.

Als Endpunkte sind die Häufigkeiten von gefäßassoziierten Veränderungen und Erkrankungen (Hypertonie, Apoplex, Infarkt) benannt.

Die statistische Auswertung soll Unterschiede in den Häufigkeiten und der Ausprägung der untersuchten Merkmale (CO, CIMT, TVR, BP, FMD, CFF, PWV, AIx) oder der chemischen Analyse (klinische Chemie und Multiplex) zeigen.

10 Datenmanagement

Alle patientenbezogenen Daten werden in pseudonymisierter Form erfasst. Dazu wird ein nicht-sprechendes Pseudonym verwendet, aus welchem allein nicht auf die Identität des Patienten geschlossen werden kann.

Die Patientenidentifikationsliste, in der die Patientenidentifikationsnummern mit den vollen Patientennamen der Teilnehmer, Patientenidentifikationsnummer und ggf. Geburtsdatum verbunden sind, dient der Möglichkeit der späteren Identifikation teilnehmender Personen. Sie ist absolut vertraulich zu behandeln und darf das Prüfzentrum nicht verlassen. Sie ist nach Studienende **mindestens zehn Jahre** zu archivieren.

Quelldaten (source data) im Sinne der ICH-GCP-Leitlinie E6 sind alle routinemäßig sowie studienbedingt erhobenen Daten. Die studienrelevanten Daten werden entsprechend den Vorgaben der Dokumentationsbögen (CRF) erhoben. Die Datenerhebung erfolgt anhand von papierbasierten Dokumentationsbögen (p-CRF). Die entsprechenden Mitarbeiter in den Prüfzentren erhalten eine Einweisung bzw. Anleitung zum Ausfüllen der papierbasierten Dokumente.

Die Anonymität der Daten im Rahmen von Auswertungen ist sichergestellt. Die Zuordnung zwischen Patient und Patienten-ID erfolgt im jeweiligen Studienzentrum durch die Patientenidentifikationsliste und wird nicht in der Datenbank abgespeichert. Es erfolgt eine regelmäßige Komplettsicherung aller Daten.

11 Datenschutz

Im Rahmen der Studie ist es erforderlich, von den Studienteilnehmern personenbezogene Daten (z. B. vollständiger Name, Initialen des Vor- und Zunamens, Geburtsdatum, Adresse) und Daten zur Behandlung und zum Krankheitsverlauf (z. B. medizinische Befunde, Behandlungsarten, verordnete Medikamente) zu erheben und zu verarbeiten. Diese Daten werden in den Prüfzentren erhoben und in pseudonymisierter Form (d. h. ohne direkten Bezug zum Patientennamen) mit Hilfe einer Patientenidentifikationsnummer elektronisch gespeichert, an die verantwortliche datenverarbeitende Stelle übermittelt und ausgewertet. Die Patientinnen erhalten diese ID zur Eingabe in die onlinebasierte Erhebung der Basisdaten, so dass diese ohne Angaben zur Person durchgeführt werden kann.

Im Falle eines Widerrufs der Einwilligung zur Studie durch den Patienten, einschließlich der weiteren Datenerhebung, werden ab dem Widerrufszeitpunkt keine weiteren Daten erhoben. Die bisher erhobenen Daten werden innerhalb der Studie weiter verwendet und ausgewertet. Bricht ein Patient nur die Studienbehandlung ab, können die für die Studie erforderlichen Daten weiterhin erhoben und verwendet werden.

Eine Einverständniserklärung ist der Patientenaufklärung beigefügt. Die Patienten sind darüber hinaus über ihre Rechte nach DSGVO aufgeklärt.

12 Ethische Belange, gesetzliche und administrative Regelungen

12.1 Deklaration von Helsinki und Gute klinische Praxis

Die Studie wird gemäß den ethischen Grundsätzen durchgeführt, die ihren Ursprung in der Deklaration von Helsinki [*Ref.*] haben. Die jeweils aktuelle Version der Deklaration wird beachtet. Die Empfehlungen der Guten Klinischen Praxis [*Ref.*], gültig seit dem 17.1.1997, werden, sofern zutreffend, berücksichtigt.

12.2 Ethik-Kommission

Das Studienprotokoll wird mit den erforderlichen weiteren Unterlagen der zuständigen federführenden Ethik-Kommission des Studienleiters mit der Bitte um Bewertung vorgelegt. Die Studie kann erst nach zustimmender Bewertung der Ethik-Kommission beginnen.

12.3 Nachträgliche Änderungen

Das Studienprotokoll ist einzuhalten. Jede vom Prüfer zu vertretende Abweichung von den vorgesehenen Untersuchungs- und Behandlungsmaßnahmen oder -zeitpunkten ist zu dokumentieren und zu begründen (z. B. Notfallmaßnahmen).

Änderungen oder Ergänzungen des Studienprotokolls können nur von der Studienleitung veranlasst und autorisiert werden. Über Änderungen des Studienprotokolls werden die federführende Ethikkommission und die Ethik-Kommissionen der beteiligten Prüfzentren informiert. Ggf. wird erneut die zustimmende Bewertung eingeholt. Bewertungspflichtige Änderungen dürfen nicht vor der Entscheidung der Ethikkommission umgesetzt werden.

12.4 Patientenversicherung/ Probandenversicherung

Da im Rahmen der Klinischen Untersuchung keine Arzneimittel bzw. Medizinprodukte zum Einsatz kommen, besteht im Rahmen der Betriebshaftpflichtversicherung des Universitätsklinikums Versicherungsschutz im Rahmen von Forschung und Lehre.

Aufgrund der Tatsache, dass die Probandinnen und Kontrollgruppen im Rahmen dieser Studie ausschließlich für Studienzwecke in das UKJ einbestellt werden, werden diese über eine Wege-Unfall-Versicherung mit Aufenthaltsrisiko abgesichert werden.

12.5 Finanzierung

Die Studie wird des DFG gesponserte Stipendium „Clinician Scientist OrganAge“ durch die IZKF, sowie über ein zusätzliches Stipendium der MGFG finanziert.

Tabelle 5: Finanzierung

Stipendium	Finanzierung	Gesamtwert in Euro
**DFG und IZKF OrganAge**	50 %-Stelle für 3 Jahre,<br>Materialkosten von 5.000 € pro Jahr	15.000
**MGFG Forschungs-stipendium**	5.000 €	5.000
**Gesamt**		20.000

12.6 Abschluss und Publikation

Ein Zwischenbericht nach 2 Jahren, sowie ein Abschlussbericht werden im Rahmen des IZKF Stipendiums angefertigt. Die Ergebnisse der Studie werden nach erfolgreichem Abschluss publiziert.

Die Veröffentlichung der Studienergebnisse erfolgt unabhängig davon, wie die Ergebnisse ausfallen.

13 Literatur

Basit, S., Wohlfahrt, J., & Boyd, H. A. (2018). Pre-eclampsia and risk of dementia later in life: nationwide cohort study. *BMJ, 363*, k4109. doi:10.1136/bmj.k4109

Black, M. H., Zhou, H., Sacks, D. A., Dublin, S., Lawrence, J. M., Harrison, T. N., & Reynolds, K. (2016). Hypertensive disorders first identified in pregnancy increase risk for incident prehypertension and hypertension in the year after delivery. *J Hypertens, 34*(4), 728-735. doi:10.1097/HJH.0000000000000855

Bowkalow, S., Schleussner, E., Kahler, C., Schneider, U., Lehmann, T., & Groten, T. (2018). Pentaerythrityltetranitrate (PETN) improves utero- and feto-placental Doppler parameters in pregnancies with impaired utero-placental perfusion in mid-gestation - a secondary analysis of the PETN-pilot trial. *J Perinat Med, 46*(9), 1004-1009. doi:10.1515/jpm-2017-0238

Bundesamt, S. (2018). *Statistisches Jahrbuch 2018*. Retrieved from

Foo, F. L., McEniery, C. M., Lees, C., Khalil, A., & International Working Group on Maternal, H. (2017). Assessment of arterial function in pregnancy: recommendations of the International Working Group on Maternal Hemodynamics. *Ultrasound Obstet Gynecol, 50*(3), 324-331. doi:10.1002/uog.17565

Garovic, V. D., Milic, N. M., Weissgerber, T. L., Mielke, M. M., Bailey, K. R., Lahr, B., . . . Miller, V. M. (2017). Carotid Artery Intima-Media Thickness and Subclinical Atherosclerosis in Women With Remote Histories of Preeclampsia: Results From a Rochester Epidemiology Project-Based Study and Meta-analysis. *Mayo Clin Proc, 92*(9), 1328-1340. doi:10.1016/j.mayocp.2017.05.030

McLaughlin, K., Zhang, J., Lye, S. J., Parker, J. D., & Kingdom, J. C. (2018). Phenotypes of Pregnant Women Who Subsequently Develop Hypertension in Pregnancy. *J Am Heart Assoc, 7*(14). doi:10.1161/JAHA.118.009595

Melchiorre, K., Sutherland, G. R., Liberati, M., & Thilaganathan, B. (2011). Preeclampsia is associated with persistent postpartum cardiovascular impairment. *Hypertension, 58*(4), 709-715. doi:10.1161/HYPERTENSIONAHA.111.176537

Milic, N. M., Milin-Lazovic, J., Weissgerber, T. L., Trajkovic, G., White, W. M., & Garovic, V. D. (2017). Preclinical atherosclerosis at the time of pre-eclamptic pregnancy and up to 10 years postpartum: systematic review and meta-analysis. *Ultrasound Obstet Gynecol, 49*(1), 110-115. doi:10.1002/uog.17367

Orabona, R., Sciatti, E., Vizzardi, E., Bonadei, I., Valcamonico, A., Metra, M., & Frusca, T. (2017). Endothelial dysfunction and vascular stiffness in women with previous pregnancy complicated by early or late pre-eclampsia. *Ultrasound Obstet Gynecol, 49*(1), 116-123. doi:10.1002/uog.15893

Peters, S. A., den Ruijter, H. M., Grobbee, D. E., & Bots, M. L. (2013). Results from a carotid intima-media thickness trial as a decision tool for launching a large-scale morbidity and mortality trial. *Circ Cardiovasc Imaging, 6*(1), 20-25. doi:10.1161/CIRCIMAGING.112.978114

Regina, C., Panatta, E., Candi, E., Melino, G., Amelio, I., Balistreri, C. R., . . . Ruvolo, G. (2016). Vascular ageing and endothelial cell senescence: Molecular mechanisms of physiology and diseases. *Mech Ageing Dev, 159*, 14-21. doi:10.1016/j.mad.2016.05.003

Regitz-Zagrosek, V., Roos-Hesselink, J. W., Bauersachs, J., Blomstrom-Lundqvist, C., Cifkova, R., De Bonis, M., . . . Group, E. S. C. S. D. (2018). 2018 ESC Guidelines for the management of cardiovascular diseases during pregnancy. *Eur Heart J, 39*(34), 3165-3241. doi:10.1093/eurheartj/ehy340

Rolnik, D. L., Wright, D., Poon, L. C. Y., Syngelaki, A., O'Gorman, N., de Paco Matallana, C., . . . Nicolaides, K. H. (2017). ASPRE trial: performance of screening for preterm pre-eclampsia. *Ultrasound Obstet Gynecol, 50*(4), 492-495. doi:10.1002/uog.18816

Schleussner, E., Lehmann, T., Kahler, C., Schneider, U., Schlembach, D., & Groten, T. (2014). Impact of the nitric oxide-donor pentaerythrityl-tetranitrate on perinatal outcome in risk pregnancies: a prospective, randomized, double-blinded trial. *J Perinat Med, 42*(4), 507-514. doi:10.1515/jpm-2013-0212

Sepulveda, C., Palomo, I., & Fuentes, E. (2017). Mechanisms of endothelial dysfunction during aging: Predisposition to thrombosis. *Mech Ageing Dev, 164*, 91-99. doi:10.1016/j.mad.2017.04.011

Steegers, E. A., von Dadelszen, P., Duvekot, J. J., & Pijnenborg, R. (2010). Pre-eclampsia. *Lancet, 376*(9741), 631-644. doi:10.1016/S0140-6736(10)60279-6

Teichert V., S. E., Markert U., Groten T. (2018). *Untersuchungen zur Wirkung von Pentaerythrityltetranitrat (PETN) auf die Dysfunktion von Endothelzellmonolayern*.

Tiralongo, G. M., Lo Presti, D., Pisani, I., Gagliardi, G., Scala, R. L., Novelli, G. P., . . . Valensise, H. (2015). Assessment of total vascular resistance and total body water in normotensive women during the first trimester of pregnancy. A key for the prevention of preeclampsia. *Pregnancy Hypertens, 5*(2), 193-197. doi:10.1016/j.preghy.2015.02.001

Valensise, H., Lo Presti, D., Gagliardi, G., Tiralongo, G. M., Pisani, I., Novelli, G. P., & Vasapollo, B. (2016). Persistent Maternal Cardiac Dysfunction After Preeclampsia Identifies Patients at Risk for Recurrent Preeclampsia. *Hypertension, 67*(4), 748-753. doi:10.1161/HYPERTENSIONAHA.115.06674

Vasapollo, B., Novelli, G. P., Gagliardi, G., Tiralongo, G. M., Pisani, I., Manfellotto, D., . . . Valensise, H. (2012). Medical treatment of early-onset mild gestational hypertension reduces total peripheral vascular resistance and influences maternal and fetal complications. *Ultrasound Obstet Gynecol, 40*(3), 325-331. doi:10.1002/uog.11103

14 Anlagen

1. Informationsmaterial zu Vicorder® und Uscom und HEPAtonorm™ Geräten
2. Patientenanschreiben, Patienteninformation, Patientenaufklärung, Fragebogen Demographische Daten
