# Supplementary material for: Cardiovascular health of women 10 to 20 years after placenta-related pregnancy diseases considering the possible effect of pentaerythrityl tetranitrate treatment during pregnancy on long-term maternal cardiovascular health (PAVA study)
Source: PLoS One. 2024 Oct 15;19(10):e0309177. doi: 10.1371/journal.pone.0309177 (PMC11478798; doi:10.1371/journal.pone.0309177)
Supplement: Attachment — Submitted filename: renamed_0fa13.docx [file pone.0309177.s005.docx]

Dear Editor and Reviewers,

we like to thank you for the valuable comments and are convinced that the manuscript is now further improved. Please find the detailed responses below.

Best Regards Tanja Groten in behalf of the authors

1. **Reviewers' comments:**

**Reviewer #1:** This single center study compared 53 participants after PE/FGR and 51 controls

That were followed up at an average of 14 years after index pregnancies. The results confirmed the previous finding that the risk of CVD is increased in women after PE/FGR compared to women after uneventful pregnancies. However, there were no differences in cardiovascular function observed, which is unexpected and contradictory to that of some previous studies. The authors also put a cautionary note on interpreting the observation that affected women with PETN intake during pregnancy showed lower mean values for right atrial area and ventricle in comparison to controls and also to affected women without former medication. Overall, it is a well carried out study with meticulous analysis.

*We thank the reviewer for this appreciative comment.*

Comments:

1) Multiple testing correction was not taken in this study. Note that the subtle changes observed for those with PETN would became insignificant if multiple testing correction procedure was taken.

*We thank the reviewer for that comment. Not using multiple testing correction is indeed a limitation. Due to the small number of events, the analyses should be seen as exploratory. This also makes it more difficult to apply multiple analysis methods, which is why we primarily used simple analysis methods. We have added a comment to this fact in the discussion (page 22, line 374-377).*

2) In the current study the age at the follow-up evaluation is with a mean of ~44-45 and range of 40 to 49. In Courntouris et al. (2021), the mean age was ~48, and in Tokslage et al. (2017) it is above 50s’. Can the difference observed between the current study and the previously study due to the age difference? A hypothesis is that the long-term effect of PE/FGR is also correlated with age of onset.

*We thank the reviewer for this thought. This could well be true, but of course it is not possible to answer this question with our data set. It could also be a fact, described by others, that the differences between groups are even greater 5 years after pregnancy and probably decrease at later time points, as we have pointed out in our discussion. However, we have added a sentence commenting on the age issue in the discussion (page 18, line 293-296).*

**Reviewer #2:** In this manuscript, the authors present interesting data from the PAVA study, with particular focus on long-term outcomes following placenta-related complications during pregnancy. The authors also conducted a sub-analysis investigating the potential role of pentaerythrityl tetranitrate in influencing prognosis. This particular topic is of interest, as far too many women face potentially life-threatening complications during pregnancy, and too few data on potential treatment strategies for these conditions are currently available. However, the results of this analysis are based on a very small sample, especially considering the number of pregnant women facing the diagnosis of pre-eclampsia each year. This becomes more evident after noticing that only crude statistical analyses could be conducted. Maybe, using these data as a companion to a meta-analysis of currently available data could allow for slightly stronger conclusions.

*We thank the reviewer for this valuable comment. We are aware that our cohort, particularly the sub-cohort taking PETN during their index pregnancy, is particularly small. For this reason, we were cautious in formulating our conclusions but were still able to point to some significant findings. However, we are aware that our data cannot be generalized at this point. Due to the small number of events, analyses are rather exploratory, which is why we primarily used simple analysis methods with individual consideration of influencing variables. We have added a comment on this limitation to the discussion (page 22, line 374-377). We are happy to take up the suggestion of conducting a meta-analysis for a future publication.*

2. **Journal requirements:**

1. Please ensure that your manuscript complies with the PLOS ONE style guide, including the file naming guidelines.

*Editing is done analogue to PLOS ONE style sheets.*

2. Thank you for stating the following financial disclosure: “This work was funded by the Deutsche Forschungsgemeinschaft (DFG, German Research Foundation) Clinician Scientist Program OrganAge funding number 413668513 and by the Interdisciplinary Center of Clinical Research of the Medical Faculty Jena to AM.” Please state what role the funders took in the study. If the funders had no role, please state: "The funders had no role in study design, data collection and analysis, decision to publish, or preparation of the manuscript." If this statement is not correct you must amend it as needed. Please include this amended Role of Funder statement in your cover letter; we will change the online submission form on your behalf.

*The requested financial information has been processed.*

3. When completing the data availability statement of the submission form, you indicated that you will make your data available on acceptance. We strongly recommend all authors decide on a data sharing plan before acceptance, as the process can be lengthy and hold up publication timelines. Please note that, though access restrictions are acceptable now, your entire data will need to be made freely accessible if your manuscript is accepted for publication. This policy applies to all data except where public deposition would breach compliance with the protocol approved by your research ethics board. If you are unable to adhere to our open data policy, please kindly revise your statement to explain your reasoning and we will seek the editor's input on an exemption. Please be assured that, once you have provided your new statement, the assessment of your exemption will not hold up the peer review process.

*All authors agreed in advance on a data sharing plan in case of paper acceptance.*

4. Please amend the manuscript submission data (via Edit Submission) to include authors Laura Bäz, MD, Thomas Lehmann, PHD, Yvonne Heimann, Ms., Ekkehard Schleußner, Prof. and Marcus Franz, Prof.

*The manuscript submission dates have been changed according to requested requirements.*

6. Please include captions for your Supporting Information files at the end of your manuscript, and update any in-text citations to match accordingly. Please see our Supporting Information guidelines for more information: http://journals.plos.org/plosone/s/supporting-information.

*Appropriate information on supplemental material has been included within the manuscript.*

7. Please review your reference list to ensure that it is complete and correct. If you have cited papers that have been retracted, please include the rationale for doing so in the manuscript text, or remove these references and replace them with relevant current references. Any changes to the reference list should be mentioned in the rebuttal letter that accompanies your revised manuscript. If you need to cite a retracted article, indicate the article’s retracted status in the References list and also include a citation and full reference for the retraction notice.

*The reference list was again verified for completeness and accuracy.*
